# Gender-Based Experiences and Perceptions after the 2010 Winter Storms in Atlantic Canada

**DOI:** 10.3390/ijerph121012518

**Published:** 2015-10-08

**Authors:** Liette Vasseur, Mary Thornbush, Steve Plante

**Affiliations:** 1 Department of Biological Sciences, Brock University, 500 Glenridge Avenue, St. Catharines, ON L2S 3A1, Canada; E-Mail: lvasseur@brocku.ca; 2 Department of Geography, Brock University, 500 Glenridge Avenue, St Catharines, ON L2S 3A1, Canada; 3 Département sociétés, territoires et développement, Université du Québec à Rimouski, 300, Allée des Ursulines, Rimouski, QC G5L 3A1, Canada; E-Mail: steve_plante@uqar.ca

**Keywords:** climate change adaptation, natural hazards, climate-based events, flooding, experiences, responses, gender mainstreaming

## Abstract

This paper conveys the findings of the first phase of a longitudinal study into climate change adaptation in Atlantic Canada. Men and women from 10 coastal communities in three provinces (Quebec, New Brunswick, and Prince Edward Island) were interviewed to better understand how both sexes perceived and reacted to extreme weather events. Their responses were recorded based on their experiences, personal and community levels of preparedness, as well as help received and effects on their lives. Most importantly, the findings denote that more men were personally prepared and more active in the community than women. More men recognized a deficiency in help at the community level, and were critical of government in particular, addressing a lack of financial interventions and support. Women were forthcoming with their emotions, admitting to feeling fear and worry, and their perceptions in terms of impacts and actions were closer to home. The results support what others have shown that in rural and coastal communities the traditional division of labor may influence and lead to a gender bias in terms of actions and gradual adaptation in communities. There is a need to better understand how these sometimes subtle differences may affect decisions that do not always consider women’s roles and experiences in the face of extreme events.

## 1. Introduction

The literature on gender, disaster management, and climate change adaptation is rather limited [1]. Nevertheless, it is known that women (and youth) can be vulnerable to extreme weather events because of their limited adaptive capacity and social-cultural discrimination, as evident within the context of post-Hurricane Katrina events in New Orleans, USA [2]. Women are also often made vulnerable due to their marginalization and reduced participation in national to international negotiations in climate change adaptation [3]. At the community level, women’s participation has been challenged due to their daily domestic activities and often lack of education. Their limited education also constrains their ability to adapt to climate change, as evident with sustainable technology (solar home systems) [4]. In several countries (mainly developing), gender inequality, which is often rooted in social practice and cultural norms, leads to major road blocks in terms of effectively communicating with authority and enabling women to adapt [4]. Milne [5] argues that in wealthier developed countries, there should be less gender differentiation in regards to disaster responses and preparedness than in developing countries.

Interactions between sex and other potential sources of (either positive or negative) discrimination, such as age, complicate matters and suggest that it should not be considered solely as binary (men *versus* women). Instead, gender should be viewed as a social category that can be compounded, as with other identities, roles, and responsibilities [6]. Some authors have also questioned whether climate change adaptation exacerbates or simply alters gender inequalities as well as other socioeconomic relations (such as inter-caste dependencies) [7]. Even though it is understood that climate change *per se* does not discriminate for sex, caste / class, age, and physical ability as well as wealth, it is recognized that it can exacerbate inequalities, causing those with poor adaptive capacity to become vulnerable [8].

In rural communities, issues of gender and poverty are often interrelated and can be more complex to understand. Rural communities, including coastal communities in Atlantic Canada, are generally vulnerable to climate change and extreme events, such as hurricanes and storm surges, due to their aging population, decline in economy (which is already precarious due to weather conditions), and now the prospect of climate change [5,9]. Most coastal communities in Atlantic Canada already feel more vulnerable than urban centers due to their geographic isolation [5], their low level of literacy, their dependency on natural resources, youth migration, and distance from policy decision-makers [10].

Social considerations of gender as well as culture are known to (even critically) affect local adaptive capacity [11]. However, gender is often an afterthought in research studies. For instance, Denton [12] posited that this is the case in the climate discussions. There has been a push to ensure gender mainstreaming in international negotiations, such as the United Nations Framework Convention on Climate Change [13]. The number of women was monitored, for instance, to assess participation at the Climate Change Congress held in March 2009 in Copenhagen [14], and the authors discovered that less than one-third of first-listed presenters in oral presentations were women. This limited representation of women and women’s issues was especially pronounced in the geosciences, where less than 20% of talks were delivered by women (compared to over 40% in the social sciences). The authors [14] suggested that: “Efforts to address gender and geographical equality, as well as forging closer links between the geosciences, the social sciences, and the humanities will greatly enhance links between knowledge and action and further help to address climate change” (p. 1001). While the number of women participating in those negotiations remains low, the acceptance of integrating gender concerns into policies has gained momentum. Yet, several steps remain to clearly comprehend the impacts of these policies on women and how gender issues are played out in local communities faced with climate change impacts. It may seem that this issue is mostly linked to developing countries; however, gender-sensitive research in developed countries has been advocated to examine gaps between policies and reality in communities. This has been the case for forest-based communities in Canada, where gender mainstreaming for effective adaptation within the context of long-term climate change effects has been included [11].

Gender-based adaptation responses to climate-related events can help to diminish women’s vulnerability [15], and gender mainstreaming is needed in local adaptation planning. In Nevada, USA, for instance, women ranchers and farmers were found to be more concerned about climate change than men, and their knowledge on the topic more scientifically accurate [16]. Research examining drought in rural Nevada has shown that age is not a significant determinant of risk perception, but that gender is important in shaping risk perception [17]. Through social interaction and experience as well as the integration of local knowledge and use of new technologies [18], women can become more resilient. Because income limits their access to resources, including new technologies, this particularly poses a key struggle for women, even in developed countries; as for instance in Adelaide, Australia, where gender and household income are the main (statistically significant) variables affecting awareness and attitudes towards heat waves associated with climate change [19].

Coastal communities are highly and increasingly vulnerable to climate change impacts, especially sea-level rise and storm surges [20]. Coastal ecosystems are fragile systems that can be dramatically changed due to the impacts of climate hazards. These changes can increase the vulnerabilities of the populations, and adaptation strategies must be developed to ensure the sustainability of these social-ecological systems. In Atlantic Canada (except for a few municipalities, such as Halifax and St. John), coastal communities are mostly rural and small agglomerations with traditional resource-based activities, such as fisheries and agriculture. Data show that the region is already affected by climate change, including increased temperatures, sea-level rise, and possibly an increase in storm-surge frequency and ocean-surface temperatures [10]. In December 2010 and January 2011, a series of storm surges occurred in the region of the southern Gulf and Estuary of St. Lawrence. Several communities were severely affected by these storms, and this led to discussion regarding how these communities could be better prepared and adapted to climate-related hazards. In Quebec (QB) alone, more than 77 communities to the east of Quebec City were declared as disaster areas and, thus, eligible for disaster relief and financial aid by the provincial government.

The purpose of this study was to examine, using a gender view, the experiences and the perceptions of men and women affected by storms in 10 coastal communities of Atlantic Canada. Broader questions encompassing storms more generally and climate change at large were also incorporated into the research in order to assess the level of previous experience. Interviews gauged the personal / household as well as community experiences of individuals.

## 2. Experimental Section

The communities covered were located in three Canadian provinces (Quebec, New Brunswick (NB), and Prince Edward Island (PEI)). Ten communities were originally selected according to the following criteria: (1) these communities were similar in terms of size (with less than 10,000 people according to Statistics Canada [21]) and (2) had not participated in climate change adaptation initiatives in the past (Table 1). These communities had been affected in the past by various storms, some more directly by a series of storms and flood episodes that occurred in December 2010 and January 2011. Storms are defined here as seasonal and inter-annual events that include tropical cyclones and hurricanes, and storm surges amplified in high tides, all of them resulting in the elevation of water [10]. These storms are generally characterized by high winds and intense precipitation (either as rain or snow).

**Table 1:** List of the 10 communities in Atlantic Canada that participated in the interviews with the types of experiences with storms.

Community	2010 Storm Damage	Population 2011 ^†^
Sainte Flavie (QC)	Yes (surges, flooding, erosion, evacuations)	919
Rivière-au-Tonnerre (QC)	No	307
Maria (QC)	Yes (flooding, surges, evacuations)	2536
Bonaventure (QC)	No	1017
Sainte-Marie-Saint-Raphael (NB)	Yes (erosion, flooding)	955
Shippagan (NB)	No	2603
Cocagne-Grande Digue (NB)	Yes	2317 + 2182
Dundas (NB)	Not as much	6282
Morell (PEI)	Yes (erosion)	313
Stratford (PEI)	No	8574

**^†^** Source of statistical information: Statistics Canada [22].

Semi-structured interviews were conducted (after research ethics approval) in the winter 2011 and spring of 2012. Seventy-four participants were interviewed and, besides demographic questions, asked about their experiences with storms (those of 2010 and previous ones), the impacts that these storms had on them personally or the community, and their level of preparedness (did they feel prepared for upcoming storms).

Interview participants were initially recruited by a process of personal and public invitations in order to engage individuals from a diversity of sectors (*i.e.*, public sector, business / economic sector, civil organization, or civilian). Additional participants were selected as needed through snowball sampling. The interviews were conducted in person in the participant’s native language (English or French), and lasted anywhere from 0.5 to 2.0 h. All interviews were audio-recorded and transcribed in their original language. Of the initial 74 participants, after transcription of the interviews, 33 men and 22 women were kept for further analysis due to either lack of information for some questions, or in some cases the fact that couples were interviewed and the impossibility of determining who stated what. Because of the small number and similarities in their responses, it was decided to combine all interviews in one analysis instead. All interviews were coded and analyzed by *Altas.ti*. Using this software, it was possible to extract categories of commonalities by sex. In the analysis, in cases where there were multiple responses, the first one was taken as the primary response, including for instance occupation; however, in cases where multiple responses were given for hazards or other experiences, these were all taken into consideration.

## 3. Results and Discussion

**Figure 1:**
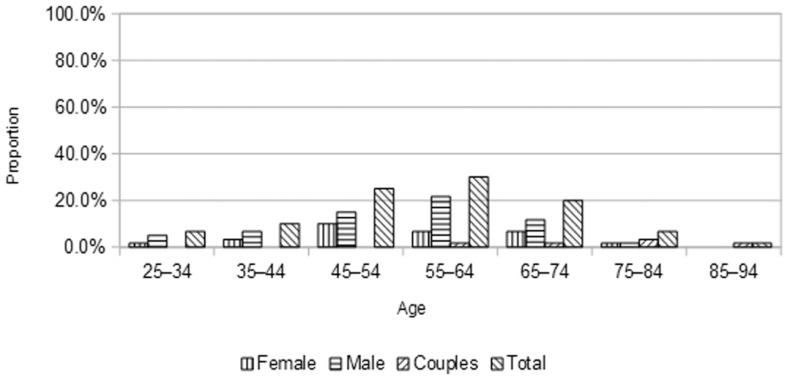
Age of participants (%) affected by storms.

All of the 33 men and 22 women were affected at some point by storms, either personally or indirectly in the community. In terms of profession, the main difference between men and women was that eight men were either fishers or farmers (no woman declared such activities). The age range of participants (Figure 1) with previous experience of storms was 26–90 years, with the most common age range for females 45–54 and slightly higher for men (55–64). It is important to note that coastal communities in Atlantic Canada represent aging populations and this sample is quite representative of the current demographics [10]. Most people interviewed have resided there most of their lives, with only two men and one woman having been in coastal communities for less than 5 years.

### 3.1. Experiences

Residents of these coastal communities experienced various storms, remembering past events in the 1970s and 1980s, 1992, 2000, and 2003–2011 as well as the “white Juan” (referring to a winter storm in 2009 similar in intensity to the 2003 Juan hurricane). Indeed, some of the participants stated that they had experienced so many storms that they could not distinguish between them in their memories. The 1990s (with the exception of 1992), however, were less mentioned in terms of frequency of storms than other decades. Hurricane Gladys (1964) was remembered as causing high tides and infrastructural damage. The aforementioned Hurricane Juan (2003) was also mentioned. People were affected by storms differently, and addressed particular storms that affected them most. For instance, one man (aged 32 years, who had always resided in NB) thought that the 2000 storm was the worst. Both men and women were able to mention these storms.

Both men and women experienced similar impacts due to storms. The hazards that were most mentioned by participants were flooding and erosion (Table 2). In general, more women (five out of seven) mentioned flooding in their home while men tended to talk about the flooding of roads and infrastructure (12 out of 15). Women tended to connect closely to personal belongings as mentioned by one “had 4 feet of water (1.2 m) in the basement. Has two pumps, but they must have stopped working. Afterwards had to throw out a lot, cut out the gyprock (wall), *etc.*”; on the other hand, men mentioned flooded roads and clogged sewage very frequently. As Fordham [23] argued, women are highly connected to their home, which is considered a gendered domain in the case of flooding; she also described the attachment that women have to their belongings in the home. Men tended to focus more on infrastructure, such as wharves, roads, and their vehicles (boats, tractors, trucks). As one man (PEI) explained: “[I] lost hydraulic winches because of storm surge (December 2010). High water went inside the wharf. Causeway started to wash away.” In most of these cases, the relation with the public work domain is very strong. In this case, the man is a fisher and as he later explained when discussing help received: “fishers help fishers.” As Davis [24] pointed out in the fishing communities of Atlantic Canada, even nowadays, the sexual division of labor can be observed, as conveyed by the current study.

**Table 2:** Summary of experience of storms. Note that the total number is greater than the number of interview participants since some might have stated more than one experience.

Effects	Female (*n* = 22)	Male (*n* = 33)
Flooding	7	15
Winds/wind storm	3	3
Heavy rain	4	3
Higher waves/tides/storm surges	0	1
Snow/blizzard	1	2
Sea ice	0	1

Erosion and loss of property were frequently mentioned by both men and women. Coastal communities in Atlantic Canada have been experiencing erosion for decades, but this has tended to increase over the past decades [25,26,27]. As one interviewee mentioned: “1990s was a period with few storms, gave a false sense of security and many people built during this time period” (man from NB). Most men who were affected by erosion mentioned the idea of building a protection wall. In addition, several who had not been affected by erosion also mentioned the existence of a protection wall. A man from NB explained that they were not really affected by previous storms and that he “believes the rock walls have helped a lot. Area is more protected than others.” Another man from NB explained that “since wharf built, there is more erosion, because currents have changed, piles up on the other side—there are dunes there didn’t used to see. 10 years ago big storm caused rocks to crumble. I know a neighbor who had to move his two chalets back from edge.” Farmers (all men) were also concerned about losing their land due to erosion.

In terms of consequences on communities, more women than men (four out of 22 *versus* two out of 33) put emphasis on power outage, while men underlined issues of damage and relocation. For example, three men mentioned having to relocate, four having lost their wharves and two their crops. Women often related damage to community infrastructure, such as community center roof collapse or the causeway. Again, this may be due to the front role of men *versus* women in these communities, where men were most likely to be outside and dealing more with hazards than women. For example, a man from PEI noted “damage to roads, damage to wharfs, road blockages, power outages. [I] worked with emergency fire department to clean up; remember time where had a hard time getting out of his driveway to go help, had to remove some trees.” On the other hand, a woman from PEI summarized the storm damage, remembering that “all the trees downed and damage from tropical Juan. After Juan had to take down two trees in the community garden in Charlottetown—they were beautiful, and gave shade, so was a real shame to take down. Really drove things home.” Two women were more concerned about the fishers than their own safety. One of the women (NB) underlined that: “Storms have had a big impact on fishers—lose traps, can’t go out, *etc.*” This relates to the traditional roles that women play in rural and coastal communities. Although many of the interviewed women were actively involved in the communities, as Davis [24] reported: “It was the task of women to worry over the safety and welfare of the family. Women’s worry was a form of women’s work and tied them into fishing ethos and occupational identity in an active and valued fashion” (p. 346). While Davis [24] related this description to fisheries in the 1970s, this mentality remains quite strong in smaller coastal communities even today (especially among older women).

### 3.2. Preparedness

In this study, more men than women felt prepared for future storms (Table 3). Interestingly, the only province where this did not hold up was PEI, where most men and women felt prepared to deal with a new storm. In most cases, they also mentioned that they had 72-h emergency supplies and very often a generator. While women frequently mentioned emergency supplies and escape / evacuation as part of their level of preparedness, men added hazard-proofing (including home and protection wall), emergency plans, and insurance. For example, a woman from PEI stated that: “If scared I’d call someone, neighbor across the street, and stay with her.” Another woman from NB felt that she was prepared by saying: “I’m resilient, have means. Would go to family members’ house.” Men tended to downplay the issue, and several made statements, such as that they “are used to living on coast” and “are used to the storms, happens often, sea rises all the time” or “It’s only temporary inconveniences—we accept what happens” (men, NB). Fishers and farmers perceived the level of preparedness in a less favorable way, stating: “Can’t predict what is going to happen 10 years forward with fishing” or “We try to be as prepared as possible, but it depends on the timing, on where you are in the crop” (men NB). Women tended to be more willing to change (*i.e.*, to make do with what is available in order to adapt [28]) when facing climate change hazards, and this gendered dimension of climate change should be integrated into planning. For example, during storms, women were more willing to evacuate than men, and this was also observed during the 2005 Katrina hurricane in the USA [29].

**Table 3:** Preparedness (personal and community) for storms. Note that some participants did not respond to these questions.

Preparedness	Female	Male
**Personal:**		
Yes	8	19
No	11	7
Uncertain	3	4
**Community: **		
Yes	5	5
No	11	15
Uncertain / Improving	0	6

Men in the current study were slightly more negative or uncertain in their assessment of community preparedness than women. In general, most men and women felt that their communities were not prepared for future storms and mostly mentioned emergency plans as well as an early warning system as a way to be prepared at the community level. Women’s social functions as social actors following crises, such as Katrina and Rita [28], convey them as caretakers looking after the procurement of food and potable water as well as safe shelter and medicine (for their families) in post-disaster situations. They are also known to procure information about emergencies and disseminate it through communication networks. Men tended to refer more to government strategies for community preparedness.

### 3.3. Receiving Help

Overall, most of those interviewed, and who answered questions related to help, mentioned not receiving any help. Men and women who received help stated receiving it from the same sources, mainly local people (friends, neighbors, family) and insurance. A man and a woman (both from NB) mentioned having “paid out of own pocket” as the damage was not covered by insurance. In terms of community help, in general, men responded more to this question and more stated that the community received no help than women (17 men *versus* four women). The negative response to this question was especially obvious among NB men, some showing their frustration. For example, one man from NB stated: “Government only responsible for roads. If you get damage on your private property, it’s your responsibility;” and another: “No—people who’ve had to move did themselves, no aid program.” It is important to note that four women admitted that they were not cognizant of whether the community received help. Of those who believed that the community received help (10 men *versus* five women), both men and women first mentioned provincial aid especially to fix the roads. The second type of help commonly mentioned was from people in the community. Men also talked about help coming from emergency workers, and this might be related to roads and infrastructure or property damage that they had to deal with. Dymén *et al*. [1] suggested that because men tend to drive more, they are more aware of issues related to transportation. The limited knowledge regarding community help among women may be due to their role closer to home. As Alston [30] stated “women are more constrained by their responsibilities” (p. 9), and this may increase their vulnerability. A man from PEI, for example, mentioned that “when the causeway washed out, it is to send the husbands to block traffic.” The traditional role of the woman at home remains quite anchored in coastal communities, as has been also denoted by others, including Christiansen-Ruffman [31].

### 3.4. Effects on People

The effects of a disaster on people can take various dimensions, from psychological to economic. In this study, the main focus was on these two aspects. In terms of psychological effects of the storms, both men and women equally mentioned being stressed. The main fear concerned lacking food and healthcare. Rural coastal communities are generally more vulnerable due to their transportation problems, and this is especially obvious during storms [9]. A man from NB reflected: “People are worried, have fears. On road, worried about what would happen if there is a fire when road is low.” While women (10 out of 13 who answered the question) mentioned they were scared and worried during and after the storms, the answers of men greatly varied. Seven out of 22 who answered the questions were also worried about storms. However, six out of 22 men mentioned that they did not need to panic. There are at least three supporting quotes of this coming from men (one from PEI and two from NB, respectively): “Typical response is to simply deal with it”; “Doesn’t feel people are stressed—said they are used to this, high winds, high tides”; and “Have seen other storms, didn’t shock.”

It is interesting that five men also mentioned the concern regarding elderly people. Several interviewees who were older than 70 years old, and others who knew elderly people, felt that the effects of storms were increasingly difficult on them. For example, a man from NB mentioned that: “Older members of the community were scared, wondering if they had to leave.” Another one told the following story: “Another was elderly, came to him panicking, wanting the mayor to do something about water near his house (but the mayor said he could not stop Mother Nature).”

The concern and fear is also greater among people closer to the shore. This was expressed by both men and women. This was especially reported in NB, where a woman stated that: “She’s not scared, used to it. They are far from water. But those who are close are probably scared;” and a man observed that: “People living right on water are more nervous.” It was also clear that fishers were more worried. A man from NB, for instance, mentioned that: “Those involved in fisheries are more worried—about needing to put boat closer to the house for example.” The division of labor among fishing communities remains strong, and their relationships with their equipment is as important as their home [24].

Interestingly, few men (4) and women (1) mentioned the financial effects of the storms directly besides insurance coverage. A woman from PEI wanted especially to: “First thing to ask is what programs does government have to help us.” The men were more direct, stating: “People are worried about how to pay for damages” (man from NB) and “Feels people are afraid—must be afraid to drop up to $45,000 down on rock walls to protect homes” (man from NB).

## 4. Conclusions 

By adopting a gender-sensitive approach in analyzing data acquired during 2011–2012 interviews in three provinces of Atlantic Canada, it was possible to highlight some important differences in the experiences of men and women to storms. A woman from NB summarized quite well the general sentiment found amidst these coastal communities: “[I am] worried about power outages and limitations that brings. Fishers are worried about their boats, equipment; they have invested their livelihood in them. Social stress of storms impacting fishers economically, and people not knowing if they can get out on the boats, how many hours they can work in the processing plants, *etc.* if storms disrupt things. People who live right on the shore [are] worried about their property, what might happen, if they will have to move. Perception that stress is not so much about the storms; people feel safe and secure in their homes. Stress is more about economic situation.” It really brings home the gendered dimension of experience with storms, with women being closer to home and concerned about men at sea. The results of this study support those of others who have found that the traditional and cultural aspects of these communities influence the way extreme events are dealt with. Women do not react and act as men do in response to storms. Men are more likely to be active and respond at the community level, and decisions made will often be related to their actions (such as the construction of protection walls). In Sweden, for instance, it has been shown that women have different perspectives on how climate change action can be executed, but have usually little power to change policies [1]. This re-emphasizes the concern of the lack of involvement of women in broader community discussions [31]. As Alston [30] stated, there is a need for more gender-sensitive social policies that provide women with greater safety closer to home. This will become increasingly important with the aging populations of coastal communities in Atlantic Canada.
